# Immunohistochemical Analysis of a Vitreous Membrane Removed from a Patient with Incontinentia Pigmenti-Related Retinal Detachment

**DOI:** 10.3390/vision4010005

**Published:** 2020-01-02

**Authors:** Márta Janáky, András Hári Kovács, Ágnes Jánossy, Dóra Török, Béla Ivanyi, Gábor Braunitzer, György Benedek

**Affiliations:** 1 Department of Ophthalmology, University of Szeged, Koranyi fasor 4-8, H-6701 Szeged, Hungary; hari.kovacs.andras@med.u-szeged.hu (A.H.K.);; 2 Department of Medical Genetics, University of Szeged, 6720 Szeged, Hungary; torok.dora@med.u-szeged.hu; 3 Department of Pathology, University of Szeged, 6720 Szeged, Hungary; ivanyi.bela@med.u-szeged.hu; 4 Laboratory for Perception & Cognition and Clinical Neuroscience, Nyírő Gyula Hospital, 1135 Budapest, Hungary; braunitzer.gabor@gmail.com; 5 Department of Physiology, Faculty of Medicine, University of Szeged, Dóm tér 10, 6720 Szeged, Hungary; benedek.gyorgy@med.u-szeged.hu

**Keywords:** incontinentia pigmenti, epiretinal membrane, immunohistochemistry, hereditary diseases

## Abstract

This is a case history of a 23-year-old woman suffering from incontinentia pigmenti (IP). The patient’s vision in the left eye started to deteriorate due to cataract progression at the age of 22, and by the age of 23, it dropped from 0.9 to 0.04. Ultrasound examination confirmed tractional vitreoretinal membranes. Vitrectomy was performed, therefore, on her left eye. The histological evaluation of vitreous membrane revealed a complex immunophenotype (positivity for glial fibrillary acidic protein (GFAP), vimentin, S-100, anti-pan cytokeratin antibody (AE/AE3), and smooth muscle-specific actin (SMA) to various extents). The right eye remained unsymptomatic throughout this course. Besides being the first to analyze the tractional vitreoretinal membrane in IP with immunohistochemical methods, this case study points out that extreme cases of asymmetric side involvement in IP do exist, even to the point of one eye being completely unsymptomatic.

## 1. Introduction

Incontinentia pigmenti (IP) (Bloch–Sulzberger syndrome) is an X-linked, dominantly inherited disease affecting approximately 1 in 50,000 newborns [1], mostly females. It is a multisystem disorder causing dermatological, dental, ocular, and neurological alterations [2]. The diagnosis can be made by either histopathologic examination of skin biopsies, or by the genetic analysis of X-chromosome mutations. Deletions comprising exons 4–10 of NF-kappa-B-essential modulator (IKBKG/NEMO) gene in Xq28 locus can be found in 80–90% of IP probands [3]. The pathognomonic cutaneous manifestations appear at birth or within a few weeks. Nearly one-third of the patients have ocular abnormalities, which are often asymmetric [4].

## 2. Case Report

The female patient was born with a birth weight of 2900 g. Right after birth, she developed antibiotic-resistant, recurrent vesicular skin lesions on the left side of her body. The diagnosis of IP was made at the third postnatal week by immunohistochemical analysis of the skin biopsy. It revealed the characteristic eosinophil granulocytic infiltration of the vesicles and the superficial layers of corium. The diagnosis was supported from her mother recalling that she had blisters on her skin of unknown origin when she was a baby. She had no other systemic manifestation, but according to the genetic analysis, she also had the same NEMO gene.

Hyperkeratotic skin lesions and faint pigmented lines were seen at the age of one. Dentitio tarda (late appearance of teeth) was the only systemic manifestation at that time. The first sign of her vision impairment appeared in 2002, at the age of six, as a mild exodeviation of the left eye. Although the best corrected visual acuity of this eye was 0.9 (the right eye had full vision), ophthalmoscopy revealed dragged optic disc and pigmented alterations on the mid-peripheral temporal retina. IP-related vitreo-retinopathy was diagnosed in her left eye, whereas it was without any alteration in her right eye.

One and a half years later, the visual acuity slightly decreased in the left eye (0.7–0.8).

**Figure 1:**
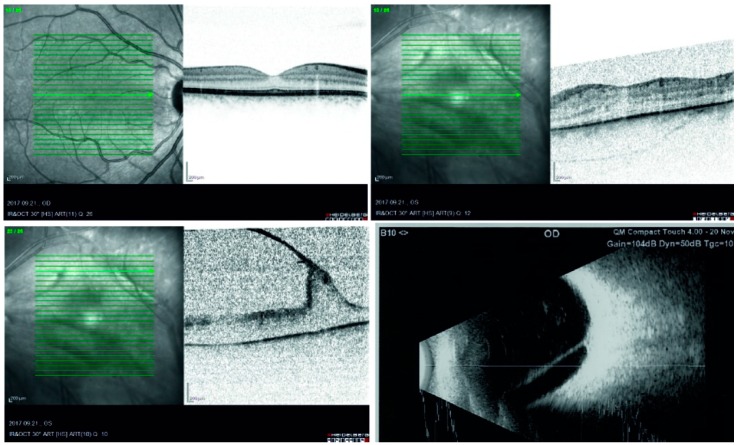
Preoperative optical coherence tomography (OCT) and ultrasound B-scans. The top left scan shows the normal macula of the right eye, and on the top right, the slightly thicker macula, flattened foveal depression, and a faint epimacular membrane of the left eye can be seen. The bottom left OCT and the bottom right ultrasound image demonstrate one of the tractional membranes in the left eye.

**Figure 2:**
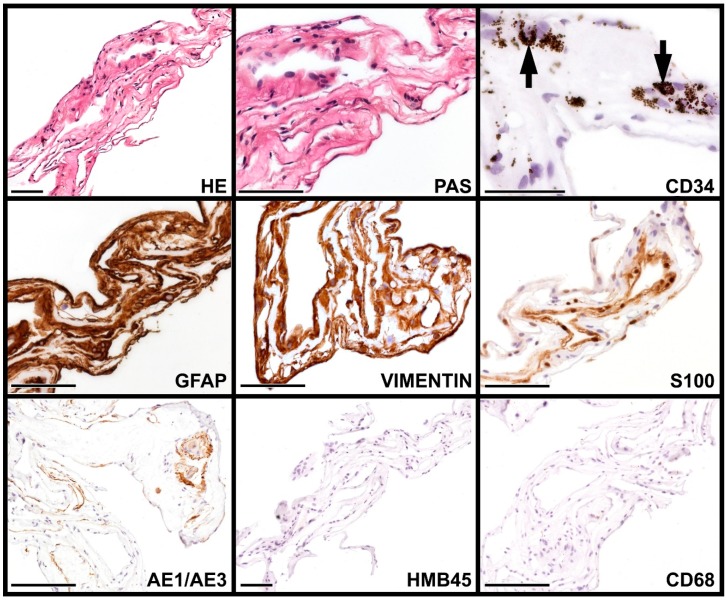
Histological and immunohistochemical features of vitreous membrane. Thin, membrane-like tissue was observed, composed of scattered cells embedded in an eosinophilic, slightly periodic acid-Schiff (PAS)-positive substance. The cells had small rounded or elongated nuclei without atypia, scanty cytoplasm, and indistinct cell borders (hematoxylin and eosin (H&E)). The cells did not rest on basement membrane (PAS). Occasionally, brownish-black pigment granules were observed in a few cells (arrows) and in the adjacent extracellular substance, shown in the cluster of differentiation 34 (CD34)-stained part. The cytoplasm of the cells and the extracellular substance strongly and diffusely reacted with GFAP and vimentin; the S-100 staining decorated the majority of nuclei and occasionally the extracellular substance. Focal cytoplasmic and extracellular substance positivity for anti-pan cytokeratin antibody (AE/AE3) and smooth muscle-specific actin (SMA; not shown) was noted. The human melanoma black-45 (HMB-45), cluster of differentiation 68 (CD68), cytokeratin 18 (CK18), cytokeratin 19 (CK19), and cluster of differentiation 34 (CD34) reactions were negative. Scale bars: 100 µm.

The patient’s left eye vision started to deteriorate rapidly due to cataract progression at the age of 22, and by the age of 23 it dropped to 0.04. Ultrasound (UH) and optical coherence tomography (OCT) revealed a tenuous epimacular membrane (Figure 1). Phacoemulsification with a single-piece posterior chamber lens implantation was performed, along with a pars plana vitrectomy. The falciform fibrotic membranes were segmented, and a small piece (1.5 × 1.5 × 0.5 mm) was submitted for morphological evaluation (Figure 2). After fixation in formaldehyde and embedding in paraffin, 3 micrometer thick tissue sections were cut and stained with hematoxylin and eosin (H&E), periodic acid-Schiff (PAS), and antibodies to glial fibrillary acidic protein (GFAP), S-100, human melanoma black- 45 (HMB-45), cytokeratin cocktail AE1/AE3 (anti-pan cytokeratin antibody), CK18, CK19 (cytokeratins 18 and 19), vimentin, clusters of differentiation 34 and 68 (CD34, CD68), and smooth muscle-specific actin (SMA). The findings revealed that the vitreous membrane had a complex immunophenotype.

In the early postoperative period, no complication occurred, visual acuity improved to 0.5, the retina was flat, and the macular contour normal. Seven days later, her best corrected visual acuity further improved to 0.6 on the operated eye.

The procedures followed were in accordance with the ethical standards of the responsible committee on human experimentation (institutional or regional) and with the Helsinki Declaration of 1975, as revised in 2000.

## 3. Discussion

The pathomechanism of retinal changes in IP remains debated. The main question to be answered is whether the retinal vascular abnormalities are primary or secondary to changes in the retinal tissue. Nishimura et al., already in 1980, suggested that arterial obstructions would lead to retinal ischaemia and proliferation [5], which was supported by Brown’s report, stating that the proliferation in IP was regulated by an ischaemia-induced angiogenic factor. Goldberg and Custis revealed peripheral retinal non-perfusion by fluorescein angiography in only 4 out of 13 IP patients [6].

Incontinentia pigmenti membrane has been investigated histologically in two IP patients so far [7,8], but neither study dealt with epiretinal membranes. An imunohistological study of the epiretinal membrane in patients with uveitis was published by Sheybani et al. [9]. In their paper, they demonstrated a mixture of abundant inflammatory cells, including lymphocytes, histiocytes, plasma cells, and occasional eosinophils, among a stromal matrix composed of glial elements and condensed vitreous, but no retinal pigment epithelium (RPE) was present. Our report describes for the first time the immunohistochemical features of the membrane in IP. Histologically, the membrane comprised sheets of cells and various amounts of extracellular matrix. Brownish-black pigment granules were seen in a few cells with diffuse positivity for GFAP, S-100, and vimentin, and focal expression of AE/AE3 pancytokeratin and SMA.

Kampik et al. described 56 cases of epiretinal membrane with differing etiology, not including IP cases. According to them, epiretinal membranes originate from retinal pigment epithelial cells, fibrous astrocytes, fibroblasts, and macrophages [10]. Edwards et al. [11] studied the preretinal membranes in age-related macular degeneration with antibodies to GFAP (marker of retinal astrocytes and activated Müller cells), vimentin, glutamine synthetase (markers of Müller cells), and ionized calcium binding adaptor molecule-1 (IBA-1; marker of microglia and hyalocytes). Both astrocytes and Müller cells were identified in the membranes. The authors have the view that stimuli in the vitreous or glial activation within the retina can induce Müller cells to migrate along the vitreoretinal surface and to recruit other cells, including astrocytes, from the retina. In another report on epiretinal membrane in age-related macular degeneration, Lee at al. [12] suggest that migration and proliferation of RPE cells and activation of glial cells occurs in response to the retinal insult.

In the study of Vinores et al. [13] on cells in vitreous culture, RPE cells expressed cytokeratin with variation in intensity, as well as vimentin, and occasionally and focally GFAP; the retinal glia expressed glutamine synthase, vimentin, and GFAP in a weak manner, and occasionally and focally cytokeratin. When grown in contact with vitreous, the transdifferentiation of RPE cells, retinal glia, and fibroblasts was observed, which led to the conclusion that the actual immunophenotype of cells is not sufficient to determine the cell type of origin of cells in epiretinal membranes. Our results support this view because the membrane in our patient exhibited a neural-glial phenotype with focal epithelial and smooth muscle cell characteristics and pigment production. The SMA-positivity could indicate the contractile property of the membrane.

Although the pronounced asymmetry in the involvement of the two eyes is well documented, the total lack of signs and symptoms of the disease in the less- or un-affected eye of our patient can be considered a rarity.
